# A systematic review of psychometric scales that assess familial, lifestyle, and behavioural factors for children living with overweight or obesity, or at risk of developing obesity

**DOI:** 10.1007/s40519-026-01860-6

**Published:** 2026-04-27

**Authors:** Bríd Áine Davis, Claire O’Dwyer, Alan Carr, Kathy Looney

**Affiliations:** School of Psychology, University College Dublin, Belfield, Dublin 4, Ireland

**Keywords:** Childhood obesity, lifestyle measures, health-related quality of life (HRQOL)

## Abstract

Over the past four decades, global childhood obesity rates have risen significantly. Modifiable factors such as diet, physical activity, family dynamics, and screen time play an important role in childhood obesity and are often targeted in interventions. However, no comprehensive review of instruments assessing these factors has been published, prompting this study. A systematic review following PRISMA guidelines was conducted to identify validated multidimensional lifestyle assessment instruments (defined as those assessing at least two constructs) for children aged 2–12 years living with overweight or obesity. Four databases (Pubmed, PsychINFO, CINAHL, and Embase) were searched for studies from 1980 to 2024. First, 7844 studies were screened by title and abstract by two independent reviewers. The resultant 141 full-text studies were also independently reviewed by 2 reviewers. Study quality was assessed and data on psychometric properties were extracted. Thirteen papers covering eight instruments were included: Healthy Kids (Niños Sanos), Family Health Behaviour Scale, Lifestyle Behaviour Checklist, Family Nutrition Physical Activity screening tool, Family Eating and Activity Habits Questionnaire, Home Environment Survey, Child Obesity Risk Questionnaire 2–5, and Energy Retention Behaviour Scale for Children. These instruments assessed various factors, including diet, mealtime routines, physical activity, sedentary behaviour, sleep, parental beliefs and the home environment. The instruments showed significant heterogeneity, with no single instrument covering the entire 2–12 years age range or measuring the same constructs comprehensively. The quality of the reviewed studies varied, particularly in the range of psychometric properties examined. Based on the results of this review, there is no one measure shown to be validated specifically for children living with overweight/obesity that addresses a broad range of domains that respond to change over time.

Level II: Evidence obtained from well-designed controlled trials without randomisation.

**Supplementary Information:**

The online version contains supplementary material available at 10.1007/s40519-026-01860-6.

## Introduction

The global prevalence of obesity has increased steadily over the past four decades, with recent data emphasising critical levels among children [13, 25]. This trend is concerning, as studies suggest that the risk of obesity in childhood continuing into adolescence and adulthood is relatively high [15]. In addition, from a public health point of view, obesity imposes a substantial burden on quality of life, encompassing various physical, mental, and social consequences, including increased risks of cardiovascular diseases, type 2 diabetes, cancers, and societal stigma [8, 32].

A wealth of literature has shown that paediatric obesity results from a complex interaction between biological, developmental, genetic, environmental, behavioural, psychological and sociological factors [5], [26]| (Dandgey and Patten [64]; Scott et al., [65]. While many of these factors are either fixed (e.g., genetic and biological factors) or impossible to influence at an individual level (e.g., the food environment and the availability of play spaces), other factors, including psychosocial, behavioural and family environment factors, can be modified and shaped, offering important avenues for intervention and prevention [55]. These factors include dietary habits, physical activity, family environment, screen time behaviours and sleep [37, 47].

Identifying the most suitable instruments for screening and monitoring relevant factors to support such interventions remains challenging. Indeed, several studies on childhood obesity interventions (e.g., [17, 48]) have resorted to using generic clinical assessment instruments—like the Child Behaviour Checklist [1]—or single-dimensional instruments—such as the Child Eating Behaviour Questionnaire [57]—rather than cohort-specific, multifactorial instruments. Results from a recent study by Koetsier et al. [28] strongly underscored the importance of using cohort-specific, user-friendly instruments that account for various factors contributing to paediatric obesity. The authors emphasised that such instruments would enhance the effectiveness of obesity management services by identifying and addressing both facilitators and barriers, which, in turn, would improve treatment success and adherence.

In addition, a review conducted by Ahuja and colleagues showed that there is currently no single instrument which fulfils the guideline criteria in terms of instrument development for assessing patient-related outcomes and psychometric validity for children and adolescents living with obesity [2]. While a recent systematic review has assessed standardised instruments for obesity-related behaviours in children aged 5 and under [9], there has not yet been a study which has focused on evaluating the psychometric properties of instruments designed specifically for preschool and school-age children (aged between 2 and 12 years) who are living with overweight or obesity.

Hence, the aim of this systematic review is to address this knowledge gap by systematically reviewing the literature to determine which familial, lifestyle, and behavioural constructs are measured in instruments designed for children (aged between 2 and 12 years) living with overweight and obesity. In addition, this review seeks to evaluate the psychometric properties of these instruments and identify their key components.

## Methods

The current review was completed in accordance with (the updated) Preferred Reporting Items for Systematic Reviews and Meta-Analysis (PRISMA) [36]. A protocol for this systematic review was registered with Prospero prior to screening of titles and abstracts (PROSPERO 2024 CRD42024517283: link).

### Databases and search strings

Based on the most common databases used in recent systematic reviews within this area [7, 9, 27, 30, 41, 42, 49, 56], the following four were searched to identify relevant studies: Pubmed, PsychINFO, CINAHL and Embase. Reference lists of relevant review articles were manually checked in addition to database searches. Field experts (including research–practitioners in obesity and public health, and child and adolescent psychology) were consulted to identify studies which may have been overlooked.

Terms used in search strings were based on terms from previous studies of lifestyle assessments of children living with overweight or obesity. Search strings were first developed using PsychINFO and then adapted to other databases. A record of the database searches is contained in Table S1 in the supplementary materials. All databases were searched by title and abstract.

### Inclusion and exclusion criteria

Lifestyle factors and well-being outcomes vary widely across the lifespan, from infancy to adolescence. Therefore, this review specifically focused on children aged 2–12 years, excluding adolescents (13–18 years), adults (18 years and older), and infants (0–2 years). Thus, the review focuses on instruments targeted at a relatively homogenous developmental stage.

In an effort to target instruments which rated lifestyle and well-being factors, this review sought instruments that were informant-rated (from the perspective of a parent or caregiver) and/or self-rated (by the child) while excluding clinician-rated instruments.

As the primary focus is on assessments for children living with overweight or obesity, included studies indicated examination of this population (in part or wholly). Studies not involving children living with overweight or obesity were excluded.

Contemporary literature (e.g., [45]) suggests that obesity and overweight in children are influenced by various factors. Hence, this review included instruments evaluating at least two constructs (e.g., dietary intake and familial/home environment factors), excluding those assessing only a single factor, such as dietary intake alone.

Only studies detailing the psychometric properties of measures (e.g., methods of item reduction, reported validity and reliability) were considered. Descriptions of analysis and results were taken directly from included studies with a commentary on whether appropriate analyses took place and what the outcomes were.

This review focused on quantitative evidence, including scale standardisation studies, experimental (randomised controlled trials, quasi-experimental trials), and observational studies (cross-sectional, case–control, and prospective studies) published in peer-reviewed journals, reports from government organisations, health agencies, and professional associations. Qualitative studies, non-English publications, book chapters, and non-peer-reviewed studies were excluded.

This review focused on studies published from January 1980, aligning with the increase in childhood obesity rates [3] to February 2024, when searches were performed. Included were English-language articles or translated versions, while unpublished studies and conference proceedings were excluded.

### Study selection and data extraction

The study selection and data extraction process involved three phases. Phase 1: After removing duplicates, all titles and abstracts were screened by two independent reviewers (BD and COD), using Covidence (https://www.covidence.org). Phase 2: Full-text screening was also conducted independently by BD and COD. In phases 1 and 2 discrepancies were resolved by consensus between the 2 reviewers and a third reviewer (AC) on a case-by-case basis. Phase 3: Data were extracted from eligible studies by BD using the template detailed in Table S2 in the supplementary material.

### Analysis plan

Due to the heterogenity of constructs assessed within the studies, a narrative synthesis was used [43], rather than a meta-analysis, b Indices of reliability and validity reported.

### Quality appraisal

Study design was evaluated using the Effective Public Health Practice Project (EPHPP) Quality Assessment Tool for Quantitative Studies [6, 50]. This tool is designed to assess various intervention study designs, such as randomised controlled trials (RCTs), pre- and post-outcome studies, and case–control studies. It examines and rates six domains (selection bias, study design, confounders, blinding, data collection methods, and withdrawals/drop-out) on a scale of Strong (3 points) to Weak (1 point). A seventh, global rating is then given, based on the six domains. Global ratings are either Strong (represented by no Weak ratings in the six domains), Moderate (only one Weak rating in the six domains) or Weak (two or more Weak ratings in the six domains). BD reviewed all studies, with 23.1% of the papers assessed independently by a second reviewer (COD) to ensure consistency. Regardless of methodological quality, all eligible studies were included to offer a comprehensive overview of the existing literature in this field. A summary of the study quality assessment is contained in Table S3 in the supplementary materials.

## Results

### Database searches and recommendations from experts

**Fig. 1:**
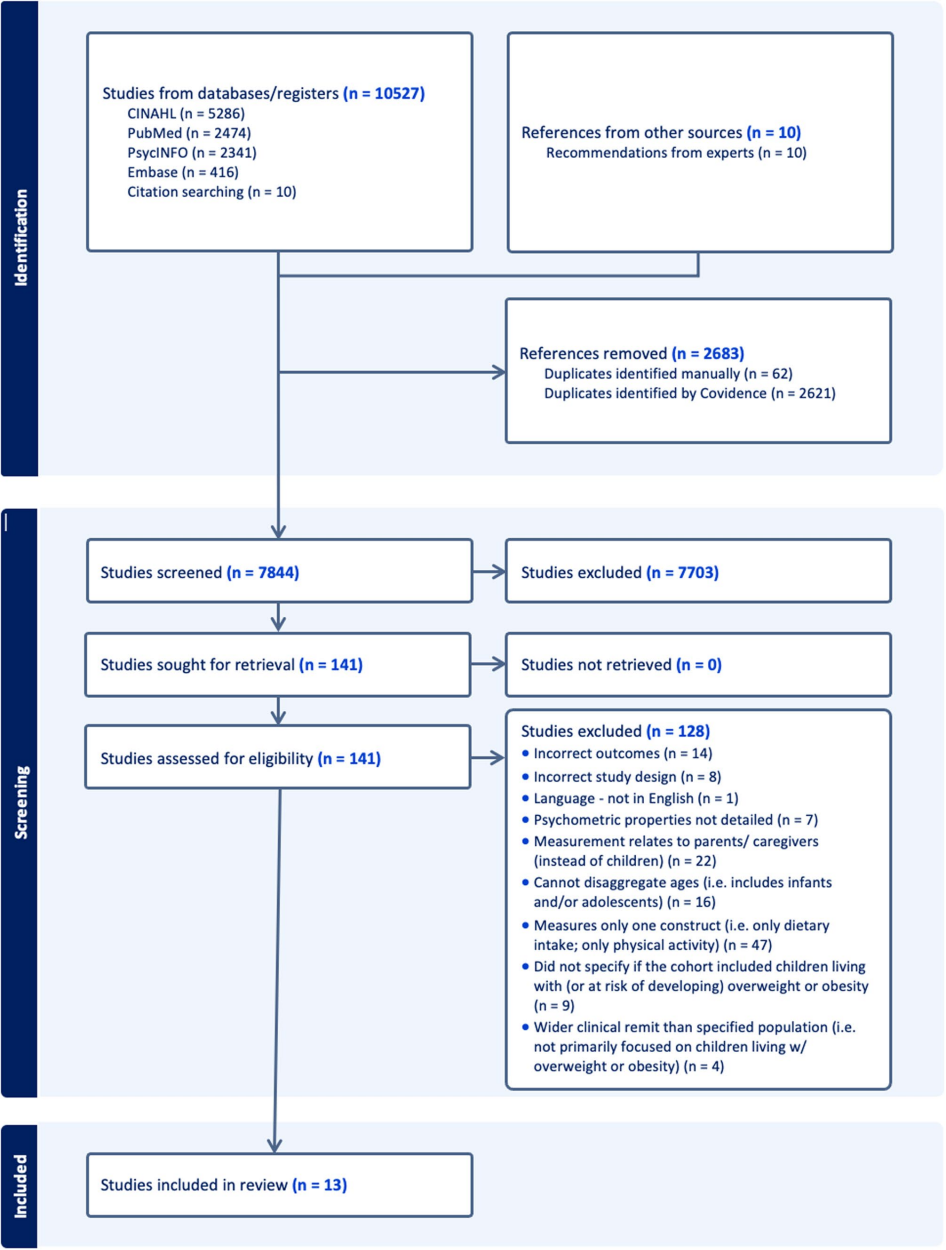
PRISMA flowchart for systematic review

Figure 1 contains a PRISMA study search flowchart; Table S4 in the supplementary materials details the PRISMA 2020 checklist [36]. The database searches identified 10,527 records, of which 7844 remained after the removal of duplicates. After screening of titles and abstracts, 7703 studies were excluded and 141 were identified as possibly relevant. A total of 10 papers were recommended by experts in the field, of which 3 were found to be relevant. Thirteen studies fulfilled the inclusion criteria and were included in the review. At this stage, the reference lists of the 13 identified papers were searched manually to identify possible omitted studies; however, no studies that met the inclusion criteria were identified.

Thus, eight instruments, assessed across 13 papers, were included for review. The instruments, along with the associated papers, are as follows. Healthy Kids/Ninos Sanos [51–53] and the Family Health Behaviour Scales (FHBS) [31, 33], [35] were each assessed by three studies. The Lifestyle Behaviour Checklist (LBC) [16, 58] was assessed by two studies. The remaining five studies were each assessed by 1 study: the Family Nutrition and Physical Activity (FNPA) screening tool [24], the Family Eating and Activity Habits Questionnaire (FEAHQ) [20], the Home Environment Survey (HES) [46], the Child Obesity Risk Questionnaire 2.5 (CORQ 2–5) [11] and the Energy Retention Behaviour Scale for Children (ERBS-C) [10]. A summary of the 8 instruments, their key properties and their strengths and weaknesses is contained in Table 1. For full psychometric data pertaining to each individual study, please see Table S5 in the supplementary material.

**Table 1:** Overview of instruments, their key properties, strengths and weaknesses

Instrument name *(Language)*	Age range	Weight status of children assessed	Instrument properties	Strengths	Weaknesses
Healthy Kids (HK)/Ninos Sanos (NS) (*English/Spanish*) [52, 53], 2023)	HK: 2–5 years NS: 3–5 years	HK: at risk of developing obesity/living with obesity (not fully detailed) NS: Underweight, normal weight, overweight and obese	HK:19 items across 2 dimensions—‘Dietary scales’ (14 items) and ‘Other Categories and scales’ (5 items) which cover parenting, screen time, physical activity and sleep NS: 18 items containing 4 subscales—eating behaviours (15 items), screen time (1 item), physical activity (1 item) and sleep (1 item) (Items differ between NS and HK with 8 new items appearing on the NS)	User-friendly with visual depictions of items Good content and face validity of items Adequate internal consistency HK shows good test–retest reliability, but this was not tested for NS For the HK, correlations within both scales were statistically significant. This was not tested for NS NS showed good criterion validity. Higher NS scores related to lower BMI and better metabolic and lipid health. Not tested for HK NS showed good convergent validity against the Healthy-Eating index total score (HEI) [29]. Not tested for HK	Item reduction (IR) for the HK was unorthodox, no factor analysis used Responsiveness was not assessed Ability of instrument to distinguish between participants in different weight categories not tested Psychometrics conducted on sample as a whole, making it difficult to link reliability/validity specifically to those with overweight/obesity Lack of precise data on weight status of participants in the HK research
Family Health Behaviour Scale (FHBS) *(English, Spanish, Turkish)* [31, 33, 35]	5–12 years	Underweight, normal weight, overweight and obese	English and Spanish versions: 27 items Turkish version: 20 items All 3 versions measure 4 factors: (1) Parent Behaviours, (2) Physical Activity, (3) Mealtime Routines, (4) Child Behaviours. Numbers of items in each subscale varies across versions	Excellent content and face validity of items Process used to develop instrument was rigorous and robust Excellent internal consistency for total and subscale scores for English and Turkish versions. For the Spanish version, Total and Parental Behaviours scores showed adequate internal consistency, while remaining subscale α scores ranged from 0.454 to 0.682 Test–retest reliability adequate for all scales for English and Turkish versions. Not tested for Spanish version Some evidence of criterion validity. BMI classification was inversely correlated with the FHBS total, Child Behaviours and Physical Activity scales for the English version. However, only the Physical Activity subscale showed this relationship in the Spanish version. Not tested in Turkish version	Responsiveness was not assessed Ability of instrument to distinguish between participants in different weight categories not tested Psychometrics conducted on sample as a whole, making it difficult to link reliability/validity specifically to those with overweight/obesity Criterion validity across many subscales in English and Spanish versions were poor. Not tested in Turkish version Ability of instrument to distinguish between participants in different weight categories not tested
Lifestyle Behaviour Checklist (LBC) *(English and Swedish)* [16, 58]	4–11 years	Normal weight, overweight and obese	English version: 26 items Swedish version: 20 items Both versions have a Confidence subscale and a Problem subscale. However, in the Swedish version, the Problem subscale is further broken down into: Overeating, Physical Activity, Emotional Correlates of Overweight, Misbehaviour in Relation to Food and Screen Time	Swedish version: Factor structure confirmed via factor analysis Subscales for both versions showed adequate to high internal consistency Test–retest reliability was acceptable for English version. Not assessed in Swedish version Both versions of the instrument could accurately distinguish between non-clinical patients and those living with overweight/obesity	English version: Factor structure not assessed Responsiveness was not assessed Majority of psychometrics conducted on sample as a whole, making it difficult to link reliability/validity specifically to those with overweight/obesity
Family Nutrition and Physical Activity (FNPA) *(English and Spanish)* [24]	Not specified. Sample included children in 1st grade only	At risk of overweight and overweight	20 items forming a single construct but covering a range of health-related behaviours (e.g., nutrition intake, sedentary behaviour, physical activity, eating habits)	FNPA factor structure was assessed by factor analysis Adequate internal consistency Significant item-total and inter-item correlations Total scores correlate with child BMI, suggesting good criterion validity	Test–retest reliability not assessed Responsiveness to change not assessed
Family Eating and Activity Habits Questionnaire (FEAHQ) *(English)* [20]	6–11 years	Normal weight children and children living with obesity	29 items across 4 domains: (1) Activity Level, (2) Stimulus Exposure (presence/visibility of treats), (3) eating related to hunger and (4) eating style. Scoring style varies significantly across domains	Good test–retest reliability FEAHQ was able to distinguish between clinical and non-clinical samples Information from multiple informants within the family Scores improved significantly following obesity intervention and also related to changes in weight, showing responsiveness to change	The factor structure was not assessed by factor analyses Internal consistency, Cronbach’s α was calculated for each subscale but not reported Item-total and inter-item correlations: not examined Complex scoring system
Home Environment Survey (HES) *(Spanish)* [46]	8–12 years	Normal weight and those with overweight/obesity	110 items. Instrument measures availability, accessibility, parental role modelling and parental policies for each of: 1.Physical Activity Resources, 2. Fruits and vegetables, 3. Sugar Sweetened Drinks and Snacks	Confirmatory factor analysis (CFA) results replicated the original four-factor structure proposed for physical activity (availability, accessibility, parental role modelling and parental policies) Multiple domains assessed	CFA did not support the factor structure of the eating habits components Internal consistency was mixed, with *α* values ranging from 0.59 to 0.84 for the Spanish version of the HES Mixed results for criterion validity. Some of the eating habits scales were significantly associated with weight status, but physical activity scales were not Test–retest reliability was not assessed Responsiveness was not assessed
Childhood Obesity Risk Questionnaire 2–5 (CORQ 2–5) *(English)* [11]	2–5 years	Children living with overweight and obesity	47 items measuring family history, child temperament, activity and eating behaviours, mealtime practices, food security, built environment, and sleep duration	Multiple domains assessed Results visually appealing	Factor structure not assessed by factor analysis Internal consistency is mixed. 4 subscales were adequate, while the remaining 4 were not Test–retest reliability not assessed Item-total correlation analysis not assessed Sensitivity not assessed
Energy Retention Behaviour Scale for Children (ERB-C) *(language not specified but instrument presented in English)* [10]	8–11 years	Underweight, normal weight, overweight and obese	17-items (reduced to 14 items) measuring (1) energy-dense food intake and (2) sedentary behaviour	CFA indicated a good model fit Each item loaded significantly to corresponding factor; standardised factor loadings ranged from 0.40 to 0.79. Moderate r = 0.42 between the 2 factors Very good internal consistency Very good test–retest reliability Instrument can accurately distinguish between clinical and non-clinical groups	Responsiveness not assessed Psychometrics conducted on sample as a whole, making it difficult to link reliability/validity specifically to those with overweight/obesity Narrow range of domains assessed

#### Healthy kids (also known as Niños Sanos)

Healthy Kids [53] is an informant-rated instrument designed to evaluate obesity risk in children aged 2–5 years, covering areas such as dietary habits, lifestyle factors, and parenting. Three studies, included in this review, investigated the psychometric properties of the 'Healthy Kids' instrument in both English and Spanish-speaking populations [52, 53] and Townsend et al. [51].

One notable strength of the Healthy Kids instrument is its item selection, which was informed by the literature, ensuring both content and face validity. The Ninos Sanos was also shown to vary according to BMI and other health markers (e.g., metabolic health). Visual depictions for items also enhance clarity, such as a child eating fruit (for a dietary intake item) or parental modelling at the dinner table (for an item on mealtime behaviour).

However, there are also issues across these studies, making it difficult to determine the overall validity and reliability of this instrument for use in clinical practice and research. As can be seen from Table 1, some psychometrics were conducted on Healthy Kids but not Ninos Sanos, and vice versa. While content and face validity of initial items were good, no factor analysis was used in the formation of the instrument. Furthermore, the content across the 2 versions varies and the precise reasons for these changes are unclear. It should also be noted that while multiple domains are assessed by these instruments, most of the items do focus on dietary habits alone.

#### The Family Health Behaviour Scale

The ‘Family Health Behaviour Scale’ [33] is a comprehensive informant-rated, multi-domain instrument designed to evaluate family eating behaviours and physical activity habits, specifically targeting children aged 5–12 years. The inclusion of multiple domains is a strength of this instrument. Validation studies of the Spanish [31] and Turkish [35] versions of the FHBS were also included in this systematic review. The FHBS was found to have many strengths. The item selection process for the FHBS [33] was thorough and involved clinical experience, evaluation of the literature, review of previous measures, input from caregivers and an expert panel.

The evaluation of the psychometric properties of the FHBS was extensive across the three studies. Factor analysis indicated a four-factor structure resulting in a 27-item measure for the English and Spanish versions [31 ,33], and a 20-item measure for the Turkish version [35]. The response format for all FHBS items was consistent, utilising the same 5-point Likert scale throughout.

However, like the Healthy Kids/Ninos Sanos, psychometric testing was carried out on the entire sample, including children ranging from underweight to living with obesity. This makes it difficult to comment on the validity of the measures specifically for those living with overweight and obesity. Furthermore, caution is advised when using this measure for pre- and post-intervention monitoring due to the lack of examination of responsiveness to change.

#### The lifestyle behaviour checklist

The Lifestyle Behaviour Checklist (LBC) [58] is an informant-rated, 26-item instrument (with 2 additional open-ended optional questions) comprised of two parallel scales which focused on paediatric obesogenic behaviours—dietary intake, screen time, physical activity—and parents’ confidence in managing these behaviours. Both the initial study by West and Sanders [58] and a study evaluating the Swedish version of the LBC were included in this review [16].

While the original English version of the instrument was informed by qualitative studies of parents’ experiences from the literature, the authors themselves generated the final list of items [58]. Thus, for the English version, the factor structure has not been confirmed. A subsequent study [59] explored the factor structure but fell outside the scope of this review as it included an age range of 4–13 years, so it was excluded. Adequate factor structure was demonstrated in the Swedish version. Indeed, the range of constructs (see Table 1) assessed is a strength of this instrument. Furthermore, a notable strength of the LBC is its focus on both problems experienced by families and parental confidence in managing these problems. Another benefit for both research and clinical practice lies in the instrument’s ability to distinguish between clinical and non-clinical samples.

However, like Healthy Kids, some psychometrics were assessed in the English version but not the Swedish version, and vice versa, meaning that neither has been robustly assessed. Furthermore, once again, responsiveness to change was not assessed and the majority of validity/reliability assessments were carried out on the sample as a whole.

#### The family nutrition and physical activity

The Family Nutrition and Physical Activity (FNPA) [24] is a 21-item informant-rated instrument designed to assess family behaviours associated with increased risk of childhood overweight. Developed by virtue of a literature review, findings from an American Dietetic Association Evidence Analysis project, statistical analysis and expert input, it covered 10 domains, including breakfast habits, modelling of nutrition, and physical activity patterns. Despite this, factor analysis suggests one underlying factor. A strength of this instrument is its criterion validity. However, while psychometric analyses suggest adequacy, the study’s cross-sectional design limited examination of test–retest reliability and responsiveness to change. Consequently, its suitability for intervention outcome monitoring remains unclear. Notably the psychometric properties of the FNPA have been scrutinised in recent years, resulting in several revisions (i.e., Peyer et al., 2017) [40] However, these studies were not included in this review as they did not meet the inclusion criteria (e.g., age range).

#### The family eating and activity habits questionnaire

The Family Eating and Activity Habits Questionnaire (FEAHQ) [20] is an informant-rated instrument used to identify multiple factors which contribute to childhood obesity and track environmental and family behaviour changes associated with weight loss. The FEAHQ assessed both family and the child’s behaviours and consisted of 4 scales: activity levels, stimulus exposure (i.e., exposure to unhealthy food), eating related to hunger and eating style (i.e., behaviours and habits, parents’ influence, etc.). Item selection was informed by an extensive literature review and pilot, and reliability studies were conducted to evaluate internal consistency, test–retest reliability, and construct validity. However, the factor structure and item-total and inter-item correlations of the measure were not examined. In addition to measuring multiple domains across informants, the FEAHQ demonstrated responsiveness to change in relation to intervention. Furthermore, these changes correlated with changes in weight.

However, while the FEAHQ assesses various domains, its scoring structure is intricate. Each item has a unique rating and scoring method, with separate scores calculated for each family member. The scoring system involves quantifying responses in hours and assigning positive or negative values based on the item. This complexity may hinder clinical adoption. Golan and colleagues conducted a more comprehensive examination resulting in the revised FEAHQ-R in 2012 (Golan [19]). However, this study was excluded from this review as it focused on parent pairs of children aged 6–16 years, falling outside the study’s scope.

#### The home environment survey—Spanish version (HES-S)

The Home Environment Survey (HES) is an informant-rated instrument originally developed by Gattshall et al. [66]. The initial validation study of HES was not included in this review as it assessed children up to 13 years, which is beyond the scope of this review. This section focuses on a subsequent validation study of the Spanish version of HES (HES-S) conducted by Sepúlveda and colleagues [46]. The HES was designed to assess how children’s weight status is influenced by two aspects of the family environment: (1) food availability and accessibility and (2) physical activity environment, which can contribute to either a healthy or unhealthy home setting. The HES-S comprised 110 items across 10 scales covering nutrition (fruit/vegetable availability, accessibility, fat/sweets availability, accessibility, parental role modelling, and policies) and physical activity (availability, accessibility, parental role modelling, and policies).

An advantage of the HES-S is its comprehensive examination of various factors contributing to obesity in paediatric populations, providing valuable information to healthcare professionals. This validation study rigorously assessed the psychometric properties of the HES-S, incorporating data from accelerometers to monitor children’s daily activity levels and additional standardised measures to evaluate convergent validity. Despite its thorough examination, the study revealed mixed results regarding HES-S’s psychometric properties, prompting a call for further validation research [46]. Therefore, two limitations of the HES identified in this study are its lengthiness and uncertainty regarding its full psychometric validity.

#### The Childhood Obesity Risk Questionnaire 2–5

The Childhood Obesity Risk Questionnaire 2–5 (CORQ 2–5) [11] is an informant-rated instrument which assesses various risk factors for obesity in children aged 2–5. It covers 8 domains, including family history, child temperament, activity and eating behaviours, mealtime practices, food security, built environment, and sleep duration, with items informed by literature and validated instruments.

One notable strength of the CORQ 2–5 is its thorough assessment across various domains, providing healthcare professionals with a comprehensive understanding of a child’s obesity risk. Moreover, it uniquely generates a visual representation of the child’s risk profile, aiding in intervention planning. However, it has some drawbacks It is the only computer-based measure in this review, which could hinder administration in clinical settings. In addition, its lengthy nature may pose time constraints for completion. Furthermore, the authors conducted minimal psychometric validation analyses, suggesting that caution is required, particularly for intervention monitoring.

#### The Energy Retention Behaviour Scale for Children (ERB-C)

The Energy Retention Behaviour Scale for Children (ERB-C), developed by Chen et al. [10], is a 17-item screening instrument which assesses obesity risk in children. It comprises two scales: energy-dense food intake (E-intake subscale, 11 items) and sedentary behaviours (S-subscale; 6 items). Chen et al. extensively evaluated the ERB-C’s psychometric properties in children with overweight and obesity.

Unlike other measures in this review, the ERB-C is completed by the child, offering direct insight into their behaviours. Its design assesses negative intake behaviours in terms of frequency, allowing clinicians to gauge the success of interventions aimed at reducing these behaviours. Likewise, the measure is brief, the language is accessible, and the checklist style is appropriate for children to complete. However, the ERB-C does not consider environmental, familial, or lifestyle influences that may provide additional context to the child’s behaviours or highlight areas for intervention. Therefore, the ERB-C is best utilised as part of a broader assessment battery alongside measures that capture these attributes, rather than as a standalone tool.

## Discussion

This systematic review aimed to assess multi-domain, psychometrically valid instruments for evaluating lifestyle, familial, and behavioural factors in children aged 2–12 years with overweight and obesity. Thirteen studies, covering eight instruments meeting the inclusion criteria, were analysed. The following section will outline: (1) an overview and critique of instruments; (2) implications for clinical practice and research; and (3) strengths and limitations of this review.

### Overview and critique of instruments

Consistent with existing literature, diet and activity levels were the two constructs most commonly included across instruments. Diet emerged as the most commonly evaluated construct for children with overweight or obesity. Some instruments, like the Healthy Kids Scale [53], explicitly quantified food intake, while others focused on mealtime routines (e.g., Family Health Behaviour Scale) and eating habits (e.g., Family Nutrition and Physical Activity Scale). Activity levels, in terms of engaging in exercise (e.g., FEAHQ) and/or sedentary behaviours (ERB-C), were also consistently measured across all instruments. Guidelines have consistently identified dietary intake, physical activity, eating habits, and sedentary behaviours as pivotal factors in paediatric obesity development [14]. In addition, interventions often target these behaviours in paediatric weight management programmes (e.g., [34]). Parental behaviours, modelling and beliefs were frequently assessed, varying from comprehensive evaluations (e.g., LBC) to more limited inquiries (e.g., Healthy Kids questionnaire). The home environment and role of the family were prominent in several (but not all) instruments, often reflected in the titles of the scales (e.g., FEAHQ, FNPA, and HES-S). Less commonly evaluated constructs included sleep and screen time usage, wherein the former was primarily assessed in measures tailored for younger children, such as the CORQ 2–5, and the latter was treated as a separate entity to sedentary behaviour (e.g., in the LBC).

While the range of dates in the review spanned from 1980 to 2024, only one of the instruments, the FEAHQ [20] predates 2009. This likely reflects the growing concern related to paediatric obesity over time. It is also likely that earlier instruments were unidimensional and, thus, would have been excluded from this review. Furthermore, the ratings of the quality assessment suggest that the general quality of studies improved over time.

No single instrument spanned the entire 2–12 years age range. Instead, some focused on early childhood (Healthy Kids: 2–5 years; CORQ 2–5: 2–5 years), others on later childhood (ERB-C: 8–11 years; HES-S: 8–12 years), and some covered middle to later childhood (LBC: 4–11 years; FHBS: 5–12 years; FEAHQ: 6–11 years). One instrument did not specify age (FNPA). This finding echoes existing literature, which highlights instruments tailored for either pre-schoolers or school-age children, possibly reflecting developmental and parenting differences between the age groups.

Seven of the eight instruments were parent or caregiver-reported, while the ERB-C was child-reported. From a content point of view, the ERB-C offered limited insights into the child’s behaviours and factors influencing them, functioning solely as a frequency measure when compared to the other instruments. Recruitment of the participants varied widely. Some participants were linked to an intervention trial, some were a sample of convenience, and some were recruited from community settings—i.e., townships and schools. Responses to questions differed widely both across and within measures.

Instruments were paper-based, except for the CORQ 2–5, which was computer-administered which, in real-world clinical practice, may pose a completion barrier for caregivers and clinicians alike, as pen-and-paper measures offer flexibility to complete off-site (for instance). Nevertheless, completion of the CORQ 2–5 by computer conveniently provided a visual risk profile of obesity risk.

In terms of language and cultural adaptability, all instruments were available in English, while some were translated into Spanish (Ninos Sanos, FHBS, FNPA, HES), Turkish (FHBS) and Swedish (LBC). In each case, a rigorous translation process took place to ensure accuracy. For all but the FNPA and HES Spanish versions, appropriate cultural equivalence testing with the target audience was also conducted with instruments being altered accordingly. For the Ninos Sanos, culturally appropriate pictures were also sought. Once translated, there is always a risk that this will impact the psychometric properties of an instrument. For the FNPA and LBC, the papers included in this review involved psychometric testing of the translated measures. However, for the Healthy Kids/Ninos Sanos and FHBS, while various psychometric properties were assessed on each language version, no one version was assessed according to all psychometrics. Thus, caution is required for these instruments as each has only been partially assessed.

### Implications for clinical practice and research

Based on this review, there is no one measure with sound psychometric properties, shown to be validated specifically for children aged 2–12 with overweight/obesity, that addresses a wide range of domains and that responds to change over time. As such, clinicians need to be judicious in their choice of measures, carefully prioritising what they want to get from their chosen measure.

For example, if the priority is to measure multiple domains associated with obesity, the FHBS and the LBC are both good options, where a rigorous and robust method of instrument development has been demonstrated. However, even though many of their psychometric properties were shown to be adequate, these analyses were conducted on samples that included a range of weight categories. Thus, it is not possible to say whether these measures are valid and reliable specifically for use with those living with overweight/obesity. Furthermore, neither has been tested for responsiveness to change, limiting their use in intervention monitoring. On the other hand, the ERB-C demonstrated a very good model of fit via factor analysis and shows good psychometric properties. However, from a clinical perspective, the range of domains it assesses (energy-dense food intake and sedentary behaviour) is very narrow and unlikely to adequately capture change from a multi-component intervention. Again, responsiveness to change was not assessed. In fact, only one instrument (FEAHQ) showed responsiveness to change. It was also shown to distinguish between clinical and non-clinical samples. However, there was limited reporting on the psychometric properties of this measure and factor structure was not assessed.

All but two of the studies utilised samples that included a range of weight categories. While this is beneficial in exploring whether an instrument can accurately distinguish between clinical and non-clinical samples, it is problematic when it comes to considering the reliability and validity of an instrument for use with children living with overweight/obesity. Only the FNPA and the CORQ2–5 were assessed in terms of their psychometric properties specifically for this cohort. While the FNPA was shown to have good criterion validity and adequate internal consistency, test–retest reliability and responsiveness to change were not assessed. For the CORQ, psychometric data were either mixed or not assessed.

Thus, measures that do assess a range of domains (e.g., LBC and FHBS) need to be assessed for responsiveness and have their psychometric properties examined within a sample living with overweight/obesity. Further research is also needed to explore the factor structure of many of these instruments. Measures that accurately assess key domains and that respond appropriately to change over time in the target audience are a basic requirement for effective research and clinical practice.

### Strengths and limitations

A main strength of this review was its exclusive focus on instruments suitable for childhood, ages 2–12 years. Although this age range might have restricted the inclusion of other pertinent instruments, considering the significant diversity in habits and behaviours spanning from infancy to childhood to adolescence, the authors found it necessary and theoretically justified to apply this criterion. An additional strength of this review is the use of independent reviewers for all studies during both the title and abstract screening and full-text screening. However, due to resource constraints, only one person extracted the data, representing a limitation of this work.

When critiquing this review it is necessary to note that research evaluating psychometrically sound instruments is limited, with related systematic reviews—such as [30]—often having broad scopes that lack specificity for children grappling with overweight and obesity exclusively. Thus, another notable strength of this systematic review lies in its dedicated focus on instruments tailored specifically for young people living with overweight/obesity. Moreover, a considerable portion of the literature in this field has relied on utilising generic instruments, resulting in essential factors being either undermeasured or overlooked. Given the paucity of reviews in this domain—despite the alarming prevalence of childhood obesity—the emphasis on reviewing psychometrically valid instruments for children aged between 2 and 12 years living with overweight or obesity in the systematic review was justified, particularly given the critical importance of early intervention in mitigating the enduring effects of these conditions.

This review endeavoured to find instruments which are psychometrically valid, as this is important to ensure sensitivity and specificity. One significant finding from this review was that the majority of instruments designed for children living with overweight and obesity are unidimensional. Indeed, a third of studies excluded during the full-text review phase (*n* = 47) related to studies of instruments which described or evaluated only one construct (i.e., only dietary intake; only physical activity).

Conversely, the inclusion criteria may have been somewhat limiting in certain respects, particularly regarding the age range, which led to the elimination of more recent scale validation studies from the outset. While this limitation prevented a more comprehensive, up-to-date evaluation of some instruments (particularly those which have been refined and revised following subsequent studies), it reflects the current landscape of paediatric obesity, where there is no established protocol for grouping age ranges together. In addition, it is not truly known if certain items included in instruments are universally applicable to older or younger cohorts.

### What is already known on this subject?

Paediatric obesity results from a complex interaction of factors. While many of these factors are either fixed (e.g., genetic predisposition) or impossible to influence at the level of the individual (e.g., the food environment), other factors, such as dietary habits, physical activity, the family environment, screen time use and sleep are modifiable at an individual level, providing appropriate targets for intervention. However, it has been highlighted within the literature that there is a ‘knowledge gap’ for cohort-specific, psychometrically valid instruments which screen and monitor these factors.

### What this study adds?

We hope that this review provides guidance to clinicians and researchers, enabling them to make informed choices and that it supports them to select appropriate instruments for their specific purposes. Based on the results of this review, there is no one measure shown to be validated specifically for children living with overweight/obesity that addresses a wide range of relevant domains and that responds to change over time. Specific recommendations for future research have been provided.

## Supplementary Information

Below is the link to the electronic supplementary material.

Supplementary file 1.

## Data Availability

No datasets were generated or analysed during the current study.
