# Supplementary material for: A systematic review of psychometric scales that assess familial, lifestyle, and behavioural factors for children living with overweight or obesity, or at risk of developing obesity
Source: Eat Weight Disord. 2026 Apr 27;31(1):56. doi: 10.1007/s40519-026-01860-6 (PMC13253707; doi:10.1007/s40519-026-01860-6)
Supplement: Supplementary file 1 [file 40519_2026_1860_MOESM1_ESM.docx]

**Supplementary Material**

**Table S1. Record of the Database Searches**

| Search String Terms | Database searched | Date searched | No. of Hits |
| --- | --- | --- | --- |
| "AB ( Overweight OR Overweight* OR Obes* OR Obesity OR obese* ) AND AB ( Child* OR Schoolchild* OR pediatri* OR paediatr* OR youth OR youths OR Youngster* OR toddler* OR Pre-school* OR Toddler ) AND AB ( Famil* OR Family-based OR parent* OR carer* OR Caregiver OR guardian* OR Parenting OR Home OR parental behav* OR child parent relation* OR Lifestyle* OR Lifestyle changes OR Lifestyle modification OR Life style* OR Health behav* OR Sedentar* OR Screen time OR Screen use OR Sleep OR Sleep hygiene OR Physical activ* OR Physical exercise OR Exercise OR Active play OR Mealtime* OR Diet* OR Nutrition ) AND AB ( Scale OR Measure OR Instrument OR Inventory OR Survey OR Psychometric* OR Assessment OR Screening ) Publication Date: 19800101-20241231; English Language; Publication Type: Clinical Trial, Corrected Article, Journal Article, Meta Analysis, Meta Synthesis, Practice Guidelines, Protocol, Questionnaire/Scale, Randomized Controlled Trial, Research, Research Instrument, Review, Standards, Systematic Review; Age Groups: Child, Preschool: 2-5 years, Child: 6-12 years, All Child; Language: English AND Apply equivalent subjects on 2024-02-25 07:37 PM" | CINAHL | 25/02/24 | 3347 |
| "AB ( Overweight OR Overweight* OR Obes* OR Obesity OR obese* ) AND AB ( Child* OR Schoolchild* OR pediatri* OR paediatr* OR youth OR youths OR Youngster* OR toddler* OR Pre-school* OR Toddler ) AND AB ( Famil* OR Family-based OR parent* OR carer* OR Caregiver OR guardian* OR Parenting OR Home OR parental behav* OR child parent relation* OR Lifestyle* OR Lifestyle changes OR Lifestyle modification OR Life style* OR Health behav* OR Sedentar* OR Screen time OR Screen use OR Sleep OR Sleep hygiene OR Physical activ* OR Physical exercise OR Exercise OR Active play OR Mealtime* OR Diet* OR Nutrition ) AND AB ( Scale OR Measure OR Instrument OR Inventory OR Survey OR Psychometric* OR Assessment OR Screening ) Publication Date: 19800101-20241231; Publication Type: All Journals; Language: English; Age Groups: Childhood (birth-12 yrs), Preschool Age (2-5 yrs), School Age (6-12 yrs); Document Type: Journal Article; Exclude Dissertations AND Apply equivalent subjects on 2024-02-25 08:01 PM" | APA PsycInfo | 25/02/24 | 2341 |
| (((Overweight[Title/Abstract] OR Overweight*[Title/Abstract] OR Obes*[Title/Abstract] OR Obesity[Title/Abstract] OR obese*[Title/Abstract]) AND (Child*[Title/Abstract] OR Schoolchild*[Title/Abstract] OR pediatri*[Title/Abstract] OR paediatr*[Title/Abstract] OR youth[Title/Abstract] OR youths[Title/Abstract] OR Youngster*[Title/Abstract] OR toddler*[Title/Abstract] OR Pre-school*[Title/Abstract] OR Toddler[Title/Abstract])) AND (Famil*[Title/Abstract] OR Family-based[Title/Abstract] OR parent*[Title/Abstract] OR carer*[Title/Abstract] OR Caregiver[Title/Abstract] OR guardian*[Title/Abstract] OR Parenting[Title/Abstract] OR Home[Title/Abstract] OR parental behav*[Title/Abstract] OR child parent relation*[Title/Abstract] OR Lifestyle*[Title/Abstract] OR Lifestyle changes[Title/Abstract] OR Lifestyle modification[Title/Abstract] OR Life style*[Title/Abstract] OR Health behav*[Title/Abstract] OR Sedentar*[Title/Abstract] OR Screen time[Title/Abstract] OR Screen use[Title/Abstract] OR Sleep[Title/Abstract] OR Sleep hygiene[Title/Abstract] OR Physical activ*[Title/Abstract] OR Physical exercise[Title/Abstract] OR Exercise[Title/Abstract] OR Active play[Title/Abstract] OR Mealtime*[Title/Abstract] OR Diet*[Title/Abstract] OR Nutrition[Title/Abstract])) AND (Scale[Title/Abstract] OR Measure[Title/Abstract] OR Instrument[Title/Abstract] OR Inventory[Title/Abstract] OR Survey[Title/Abstract] OR Psychometric*[Title/Abstract] OR Assessment[Title/Abstract] OR Screening[Title/Abstract]) Filters: Clinical Study, Clinical Trial, Comparative Study, Controlled Clinical Trial, Evaluation Study, Guideline, Introductory Journal Article, Meta-Analysis, Multicenter Study, Observational Study, Practice Guideline, Pragmatic Clinical Trial, Randomized Controlled Trial, Review, Systematic Review, Validation Study, English, Child: birth-18 years, Preschool Child: 2-5 years, Child: 6-12 years, from 1980 - 2024 | PUBMED | 25/02/24 | 2474 |
| (overweight:ab OR overweight*:ab OR obes*:ab OR obesity:ab OR obese*:ab) AND (child*:ab OR schoolchild*:ab OR pediatri*:ab OR paediatr*:ab OR youth:ab OR youths:ab OR youngster*:ab OR toddler*:ab OR 'pre school*':ab OR toddler:ab) AND (famil*:ab OR 'family based':ab OR parent*:ab OR carer*:ab OR caregiver:ab OR guardian*:ab OR parenting:ab OR home:ab OR 'parental behav*':ab OR 'child parent relation*':ab OR lifestyle*:ab OR 'lifestyle changes':ab OR 'lifestyle modification':ab OR 'life style*':ab OR 'health behav*':ab OR sedentar*:ab OR 'screen time':ab OR 'screen use':ab OR sleep:ab OR 'sleep hygiene':ab OR 'physical activ*':ab OR 'physical exercise':ab OR exercise:ab OR 'active play':ab OR mealtime*:ab OR diet*:ab OR nutrition:ab) AND (scale:ab OR measure:ab OR instrument:ab OR inventory:ab OR survey:ab OR psychometric*:ab OR assessment:ab OR screening:ab) AND ([cochrane review]/lim OR [systematic review]/lim OR [meta analysis]/lim OR [controlled clinical trial]/lim OR [randomized controlled trial]/lim) AND ([article]/lim OR [article in press]/lim OR [review]/lim OR [preprint]/lim) AND [english]/lim AND ([child]/lim OR [preschool]/lim OR [school]/lim) AND [embase]/lim AND [1980-2024]/py | EMBASE | 25/02/24 | 416 |

**Table S 2. Psychometric Properties Extraction Guide**

| **Criterion Number** | **Psychometric Properties** |
| --- | --- |
| 1 | Scale name and acronym |
| 2 | The constructs assessed |
| 3 | Respondents - child self-report, parent report, multi-informant i.e. child and parent report versions |
| 4 | Total number of items |
| 5 | Number of subscales, if any |
| 6 | Subscale names |
| 7 | Number of items in each subscale |
| 8 | Response format e.g. 2 point (yes/no), 5 point (strongly agree to strongly disagree), |
| 9 | Age-range for which the measure is suitable |
| 10 | Sample used for validation the scale (e.g. school children, paediatric service patients, mental health service users), |
| 11 | Internal consistency (alpha) reliability coefficients of the scale and subscales, |
| 12 | Test-retest reliability coefficients of the scale and subscales, |
| 13 | If the subscales were identified by factor analysis |
| 14 | Correlation coefficients between the scale/subscales and other measures, |
| 15 | Effect sizes (*d* or *g*) reflecting differences between clinical and non-clinical groups means on the scale/subscales, or means and standard deviations of clinical and non-clinical groups which may be used to calculate effect sizes |
| 16 | Effect sizes (d or g ) reflecting differences between pre and post-intervention means indicating sensitivity of the scale and its subscales to change, or pre and post-intervention means and standard deviations which may be used to calculate effect sizes |
| 17 | Languages the scale has been translated into |

**Table S 3. EPHPP Quality Assessment Summary Table**

| **Author, year** | **Selection Bias** | **Study Design** | **Confounders** | **Blinding** | **Data Collection Methods** | **Withdrawals and Drop-Outs** | **Global Rating** |
| --- | --- | --- | --- | --- | --- | --- | --- |
| Chen et al., 2015 | Weak | Moderate | Weak | Weak | Moderate | Not applicable | Weak |
| Davies et al., 2015 | Weak | Moderate | Weak | Weak | Strong | Not applicable | Weak |
| Ek et al., 2015 | Weak | Moderate | Strong | Weak | Strong | Not applicable | Weak |
| Golan and Weizman, 1998 | Moderate | Moderate | Moderate | Weak | Weak | Not applicable | Weak |
| Ihmels et al., 2009 | Weak | Moderate | Weak | Weak | Weak | Not applicable | Weak |
| Lanzarote-Fernández et al., 2019 | Weak | Weak | Weak | Weak | Moderate | Not applicable | Weak |
| Moreno et al., 2011 | Weak | Weak | Weak | Weak | Weak | Not applicable | Weak |
| Öztürk et al., 2023 | Moderate | Moderate | Moderate | Moderate | Moderate | Not applicable | Strong |
| Sepúlveda et al., 2021 | Moderate | Moderate | Moderate | Moderate | Moderate | Not applicable | Strong |
| Townsend et al., 2018 | Moderate | Moderate | Moderate | Weak | Moderate | Moderate | Moderate |
| Townsend et al., 2020 | Moderate | Moderate | Moderate | Weak | Moderate | Moderate | Moderate |
| Townsend et al., 2023 | Moderate | Moderate | Moderate | Weak | Moderate | Moderate | Moderate |
| West and Sanders, 2009 | Moderate | Moderate | Strong | Strong | Strong | Not applicable | Strong |

**Figure S1. PRISMA Flowchart for Systematic Review**


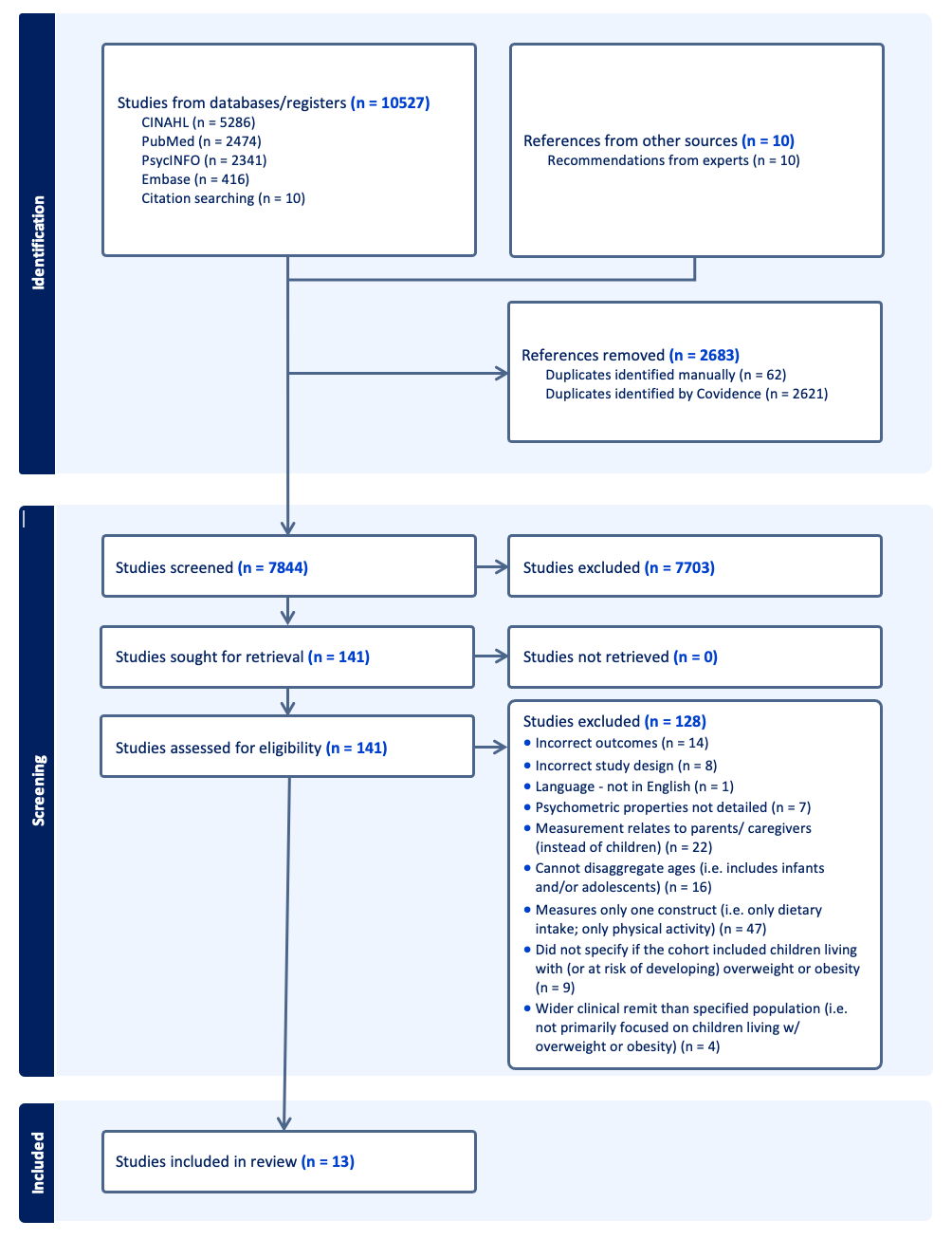


**Table S 4. PRISMA Checklist for Systematic Review**

| **Section and Topic** | **Item #** | **Checklist item** | **Location where item is reported** |
| --- | --- | --- | --- |
| **TITLE** | | |  |
| Title | 1 | Identify the report as a systematic review. | Pg 1; Title |
| **ABSTRACT** | | |  |
| Abstract | 2 | See the PRISMA 2020 for Abstracts checklist. | Pg 2; abstract |
| **INTRODUCTION** | | |  |
| Rationale | 3 | Describe the rationale for the review in the context of existing knowledge. | Pg 3-4 (Introduction) |
| Objectives | 4 | Provide an explicit statement of the objective(s) or question(s) the review addresses. | Pg 4 (Introduction; final paragraph) |
| **METHODS** | | |  |
| Eligibility criteria | 5 | Specify the inclusion and exclusion criteria for the review and how studies were grouped for the syntheses. | Pg 4 (first paragraph in method section) |
| Information sources | 6 | Specify all databases, registers, websites, organisations, reference lists and other sources searched or consulted to identify studies. Specify the date when each source was last searched or consulted. | ‘Search strategy’ section; page 5-6 |
| Search strategy | 7 | Present the full search strategies for all databases, registers and websites, including any filters and limits used. | Table S1 in supplementary materials |
| Selection process | 8 | Specify the methods used to decide whether a study met the inclusion criteria of the review, including how many reviewers screened each record and each report retrieved, whether they worked independently, and if applicable, details of automation tools used in the process. | Study Selection and Data Extraction section (page 6) |
| Data collection process | 9 | Specify the methods used to collect data from reports, including how many reviewers collected data from each report, whether they worked independently, any processes for obtaining or confirming data from study investigators, and if applicable, details of automation tools used in the process. | Study Selection and Data Extraction section (page 6) |
| Data items | 10a | List and define all outcomes for which data were sought. Specify whether all results that were compatible with each outcome domain in each study were sought (e.g. for all measures, time points, analyses), and if not, the methods used to decide which results to collect. | ‘Search Strategy’ section (pg. 5) and ‘Study Selection and Data Extraction’ section (page 6) |
|  | 10b | List and define all other variables for which data were sought (e.g. participant and intervention characteristics, funding sources). Describe any assumptions made about any missing or unclear information. | ‘Search Strategy’ section (pg. 5) and ‘Study Selection and Data Extraction’ section (page 6) and ‘Database Searches and Recommendations from Experts’ section |
| Study risk of bias assessment | 11 | Specify the methods used to assess risk of bias in the included studies, including details of the tool(s) used, how many reviewers assessed each study and whether they worked independently, and if applicable, details of automation tools used in the process. | ‘Quality Appraisal’ section |
| Effect measures | 12 | Specify for each outcome the effect measure(s) (e.g. risk ratio, mean difference) used in the synthesis or presentation of results. | N/a |
| Synthesis methods | 13a | Describe the processes used to decide which studies were eligible for each synthesis (e.g. tabulating the study intervention characteristics and comparing against the planned groups for each synthesis (item #5)). | ‘Study Selection and Data Extraction’ section; Table S2 |
|  | 13b | Describe any methods required to prepare the data for presentation or synthesis, such as handling of missing summary statistics, or data conversions. | N/a |
|  | 13c | Describe any methods used to tabulate or visually display results of individual studies and syntheses. | Table S2 |
|  | 13d | Describe any methods used to synthesize results and provide a rationale for the choice(s). If meta-analysis was performed, describe the model(s), method(s) to identify the presence and extent of statistical heterogeneity, and software package(s) used. | ‘Study Selection and Data Extraction’ section; Table S2 in the supplementary materials |
|  | 13e | Describe any methods used to explore possible causes of heterogeneity among study results (e.g. subgroup analysis, meta-regression). | N/a |
|  | 13f | Describe any sensitivity analyses conducted to assess robustness of the synthesized results. | N/a |
| Reporting bias assessment | 14 | Describe any methods used to assess risk of bias due to missing results in a synthesis (arising from reporting biases). | ‘Quality Appraisal’ section; |
| Certainty assessment | 15 | Describe any methods used to assess certainty (or confidence) in the body of evidence for an outcome. | N/a |
| **RESULTS** | | |  |
| Study selection | 16a | Describe the results of the search and selection process, from the number of records identified in the search to the number of studies included in the review, ideally using a flow diagram. | Study summaries: pages 16 – 21; ‘Database Searches and Recommendations from Experts’ section; PRISMA study search flowchart; Table S4 in the supplementary materials |
|  | 16b | Cite studies that might appear to meet the inclusion criteria, but which were excluded, and explain why they were excluded. | N/a |
| Study characteristics | 17 | Cite each included study and present its characteristics. | Table 1. Summary of Key Findings from Systematic Review |
| Risk of bias in studies | 18 | Present assessments of risk of bias for each included study. | Table S3 in the supplementary materials |
| Results of individual studies | 19 | For all outcomes, present, for each study: (a) summary statistics for each group (where appropriate) and (b) an effect estimate and its precision (e.g. confidence/credible interval), ideally using structured tables or plots. | Table 1. Summary of Key Findings from Systematic Review |
| Results of syntheses | 20a | For each synthesis, briefly summarise the characteristics and risk of bias among contributing studies. | Table 1. Summary of Key Findings from Systematic Review |
|  | 20b | Present results of all statistical syntheses conducted. If meta-analysis was done, present for each the summary estimate and its precision (e.g. confidence/credible interval) and measures of statistical heterogeneity. If comparing groups, describe the direction of the effect. | Table 1. Summary of Key Findings from Systematic Review |
|  | 20c | Present results of all investigations of possible causes of heterogeneity among study results. | Table 1. Summary of Key Findings from Systematic Review |
|  | 20d | Present results of all sensitivity analyses conducted to assess the robustness of the synthesized results. | N/a |
| Reporting biases | 21 | Present assessments of risk of bias due to missing results (arising from reporting biases) for each synthesis assessed. | Table 1. Summary of Key Findings from Systematic Review |
| Certainty of evidence | 22 | Present assessments of certainty (or confidence) in the body of evidence for each outcome assessed. | N/a |
| **DISCUSSION** | | |  |
| Discussion | 23a | Provide a general interpretation of the results in the context of other evidence. | ‘Key features of the Measures, Strengths and Weaknesses’ section |
|  | 23b | Discuss any limitations of the evidence included in the review. | ‘Strengths and limits’ section |
|  | 23c | Discuss any limitations of the review processes used. | ‘Strengths and limits’ section |
|  | 23d | Discuss implications of the results for practice, policy, and future research. | ‘Strengths and limits’ section; ‘What this study adds?’ section |
| **OTHER INFORMATION** | | |  |
| Registration and protocol | 24a | Provide registration information for the review, including register name and registration number, or state that the review was not registered. | Detailed in ‘Inclusion and Exclusion Criteria’ section; registered with Prospero (PROSPERO 2024 CRD42024517283). |
|  | 24b | Indicate where the review protocol can be accessed, or state that a protocol was not prepared. | Link provided in ‘Inclusion and Exclusion Criteria’ section |
|  | 24c | Describe and explain any amendments to information provided at registration or in the protocol. | N/a |
| Support | 25 | Describe sources of financial or non-financial support for the review, and the role of the funders or sponsors in the review. | Detailed in ‘declarations’ section |
| Competing interests | 26 | Declare any competing interests of review authors. | Detailed in ‘declarations section’ |
| Availability of data, code and other materials | 27 | Report which of the following are publicly available and where they can be found: template data collection forms; data extracted from included studies; data used for all analyses; analytic code; any other materials used in the review. | Supplementary materials |

*From:*  Page MJ, McKenzie JE, Bossuyt PM, Boutron I, Hoffmann TC, Mulrow CD, et al. The PRISMA 2020 statement: an updated guideline for reporting systematic reviews. BMJ 2021;372:n71. doi: 10.1136/bmj.n71
